# Supplementary material for: Histological and molecular characterization of the digestive system of early weaned juveniles of Arapaima sp. reared in a recirculating aquaculture system
Source: PLoS One. 2025 May 14;20(5):e0323012. doi: 10.1371/journal.pone.0323012 (PMC12077701; doi:10.1371/journal.pone.0323012)
Supplement: Attachment — Submitted filename: Comments to Authors PONE-D-24-43490.doc [file pone.0323012.s004.doc]

**Comments to the Authors**

I have reviewed your manuscript titled **"Histological and molecular characterization of the digestive system of early weaned juveniles of *Arapaima sp.* reared in a recirculating aquaculture system"** and would like to offer constructive feedback. I appreciate your effort and dedication and look forward to helping you enhance the quality of your work. Below, I will outline several points that require further consideration and improvement.

1. Abstract, Lines 23-29: Please delete or summarize the background information and proceed directly to the aim of the study.
2. Abstract, Lines 30-35: Please merge the aim of the study to avoid repetition.
3. Abstract: Please include specific recommendations in the conclusion to enhance clarity.
4. Abstract: In conclusion, please discuss your results and suggest directions for future studies.
5. Could you please provide the keywords for this study?
6. Introduction, Lines 96-112: The introduction is overly lengthy, and the aim is expressed in too many words. Please revise to clearly emphasize the aim of the study.
7. Lines 129 and Table 1: The value of crude lipid differs between the text and Table 1. Please verify and correct this discrepancy.
8. Did you use any anesthetics in the experiment?
9. Table 1: All full names of abbreviations, such as gene names, MUST be defined in the footnotes.
10. Line 261: Please italicize “(*P* < 0.05)”. Additionally, check the entire manuscript for consistent formatting.
11. I have noticed a few minor errors and spelling mistakes scattered throughout the manuscript. To enhance the linguistic quality of the manuscript, I would recommend considering professional language revision.
