# Supplementary material for: Histological and molecular characterization of the digestive system of early weaned juveniles of Arapaima sp. reared in a recirculating aquaculture system
Source: PLoS One. 2025 May 14;20(5):e0323012. doi: 10.1371/journal.pone.0323012 (PMC12077701; doi:10.1371/journal.pone.0323012)
Supplement: Attachment — Submitted filename: Response to Reviewers.docx [file pone.0323012.s005.docx]

**PONE-D-24-43490**

**Histological and molecular characterization of the digestive system of early weaned juveniles of *Arapaima* sp. reared in a recirculating aquaculture system**

**PLOS ONE**

**Replies to reviewer comments**

We sincerely appreciate the time and effort of both reviewers in evaluation our manuscript. Their constructive feedback has helped us improve the clarity and quality of our study. Below, we provide detailed responses to each comment and outline the revisions made in the manuscript accordingly.

**Reviewer #1: There are no recommendations**

**The most descriptive methodologies are without any references --why/how**

Response: We have added additional references where necessary to support the methodological descriptions. However, some methods are widely used, while others follow manufacture’s protocols (e.g., molecular biology techniques), which do not usually require specific citations.

**There are no declaration of conflict interests**

Response: We are unsure which information is visible to the reviewers, but a competing interests’ statement was provided during submission, following the instructions of the journal. The authors have declared that no competing interests exist.

**There are no authors contributions**

Response: Author contributions were also included in the online submission in accordance with journal guidelines.

**There are no data availability**

Response: The required data availability statement was also provided during submission, indicating that all relevant data are included within the manuscript and its Supporting Information files.

**There is no ethical approval statement**

Response: This information was included in the original manuscript (L168-171; L146-149 in the revised version) and provided online during submission. At the time of the study, no local ethical committee existed at the IIAP in Iquitos, Peru, the country’s main Amazonian aquaculture research institution. However, as part of a Peruvian-French-Spanish collaboration, all animal procedures adhered to the European Union Council guidelines (2010/63/EU). Compliance was ensured by the lead author, who is certified in animal experimentation by the French Ministry of Agriculture and Fisheries.

**Materials and methods very long and repeated --for example---LN/173---after the authors mentioned the histological analysis back to write it and start to mention again**

Response: We reviewed and reorganized the manuscript to eliminate unintended repetition. The “Sampling and growth measurements” section originally described sample preservation methods for the subsequent histological and gene expression analyses (LL155-160 of the original submission), while the “Histological analyses” section detailed the specific analytical techniques. However, we have revised these sections for improved clarity.

**There are no reference for the statistical analysis?**

Response: We have added a reference.

**LN/258—write as statistical analysis not else with an updated references**

Response: The section title has been updated to “Statistical analyses,” as suggested.

**Results and discussion --going to be chapter like**

Response: The meaning of this comment is unclear. However, we have carefully revised both sections and have also shorten the discussion considerably.

**Abstract:**

**There are no highlights**

Response: PLOS ONE guidelines do not require highlights.

**There are no graphical abstract**

Response: Similarly, PLOS ONE guidelines do not request a graphical abstract.

**The keywords should be written after the abstract not before**

Response: Keywords were provided during submission and not in the manuscript, as per journal requirements. Their placement in the reviewer’s version may depend on how the submission system displays them.

**LN/24-25--clarify the differences between the monotypic genus and multiple species**

Response: A monotypic genus contains a single species, unlike other genera that contain multiple species. This is standard terminology that typically does not require further explanation.

**LN/26---tendencies---detailed this**

Response: This tendency refers to plant ingestion. However, this information is no longer present in the revised abstract.

**LN/33-36--more details are requested**

Response: We revised the abstract to include additional detail within the 300-word limit.

**There are many things related to the introduction and materials and methods ---should not included at the abstract**

Response: The abstract has been revised based on the reviewer’s feedback.

**Divide the abstract into backgrounds-aims/methods/results and conclusion**

Response: PLOS ONE guidelines (PLOS ONE style templates) do not require sectioned abstracts. We have structured the abstract according to their format: “The Abstract should: i) Describe the main objective(s) of the study; ii) Explain how the study was done, including any model organisms used, without methodological detail; iii) Summarize the most important results and their significance; iv) Not exceed 300 words.”

**Add carnivores /aquaculture industry / food quality , histology and molecular analysis to the keywords**

Response: It is generally recommended that keywords avoid duplicating words already used in the title. Therefore, we don’t believe that “histology and molecular analysis” should be added to the keywords. We have replaced “early weaning” with “Peruvian aquaculture”, to align with the reviewer’s recommendation (aquaculture industry) while maintaining a geographical focus. For the rest, we opted to retain our original keywords, as they accurately reflect the study’s focus and complement the title, such as “paiche” (common name of the species in Peru), “Amazon basin” (natural habitat), “digestive gene expression,” and “ontogeny.” Furthermore, this paper does not focus on “carnivores” (we are actually showing that our species has characteristics of an omnivorous species) or “food quality”.

**Introduction:**

**LN/50---determine the time to reach**

Response: These values are historical maximums observed in nature, which are less frequently observed today due to overfishing. The time necessary to achieve them is not well-documented, although it is thought to be between 4 and 7 years. However, we prefer not to include this information as it is not well-supported. Many papers on *Arapaima* spp. mention these historical data in a similar way to us.

**LN/51--why R is capital with river**

Response: “Essequibo River” is often capitalized as a formal name, but we have changed it to lowercase.

**LN/58---what is this ?**

Response: We have defined CITES as the Convention on International Trade in Endangered Species of Wild Fauna and Flora.

**LN/59-60---2 different style in writing the references**

Response: Schinz 1822 is a species authority notation, not a reference. Parentheses indicate reclassification under a different genus than originally described.

**Introduction is extremely very long , repeated without any need**

Response: The introduction has been streamlined to improve clarity and conciseness.

**Aims need to be more clarified**

Response: The aims have been reformulated to address the comments of both reviewers. The second reviewer requested a more synthesized description of the aims.

**Novelty of this study should be more highlighted**

Response: We have emphasized the study’s novelty in the introduction.

**Materials and methods**

Overall comment: The line numbers indicated by the reviewer in the next six comments do not correspond to the lines of the Material and Methods section. Where possible, we have identified and addressed the comments accordingly.

**LN/64--write as materials and methods not VS**

Response: We were unable to identify what this refers to.

**LN/66--prepared--means what and add reference**

Response: The term “prepared” is only used once in the paper in the L 176 of the original submission (“Paraffin blocks were prepared in an AP280-2Myr station”). We are not sure what the reviewer requires. Paraffin blocks are prepared by embedding the samples in paraffin, as described in the previous sentence. This is a standard procedure in sample preparation for histological analysis. The reference “Sarasquete and Gutiérrez, 2005”, which was inadvertently not formatted correctly, was already included and we have added an additional one.

**LN/69-87---add reference**

Response: If the previous comment regarding L 66 actually refers to L 176 in the initial submitted version, we assume that the reviewer may be referring to LL 179-197. If that is the case, we have added a reference in LL 159 and 174 to complement those that were already included in LL 162, 164, and 177.

**LN/73---why empty**

Response: We could not locate the term “empty” in the manuscript.

**LN/88-98--also without any references**

Response: If the reviewer refers to the “Partial mRNA amplification and identification” section, we have added reference (LL 198 and 206). However, we would like to highlight that, as mentioned in LL 184, 188, and 197, this section often follows manufacture’s protocols.

**LN/99-106--add reference**

Response: If the reviewer refers to the “Partial mRNA amplifcation and identification” section, references have been included in LL 227 and 233.

**The authors did not mention any thing about the statistical analysis**

Response: We are not sure what the reviewer refers to. The statistical analyses were described in the original LL 258-263. However, we have made several modifications (LL 242-249).

**Materials and methods :**

**What about the IACUC code --should be enclosed**

Response: As previously mentioned, animal experimental procedures are documented in LL146-149 and were also included in the submission process.

**LN/115-123---add reference**

Response: We have added relevant references in L 104 regarding the taxonomic uncertainties (which were already provided in the introduction). However, for the rest of the text, we cannot add references as it refers to our specific rearing conditions.

**LN/124--write as a separate item ---experimental design**

Response: Given that the description of our experimental design is concise, we believe presenting the information in a single subsection is more appropriate.

**How many fishes did you use/groups**

Response: The manuscript provides details on fish density and the water volume per tank. Specifically, 1 ind L^-1^ in 30 L = 30 fish per tank (LL 106-108).

**LN/135-143----very long --no need**

Response: Diet composition details are essential in aquaculture nutrition for reproducibility and comparisons with similar studies. Therefore, we cannot remove this information.

**LN/155---you should also write as a separate items--as histological analysis**

Response: We have integrated this text into the histological analysis section (LL 152-177), although it referred to sample preservation during experimentation rather than the analytical methods themselves.

**Regarding the histological analysis ---complete details about the paraffin embedding technique should be enclosed with an updated references**

Response: We have added some additional details; however, paraffin embedding is a very standard procedure that typically does not require further details or updated references.

**Results :**

**Huge number of abbreviations were used---tabulate all as general**

Response: The only abbreviations used in the main text of the results are TL (total length) and WW (wet weight), which are defined upon their first mention in the Introduction and Material and Methods sections, respectively (L 60 and L136), as well as gene name abbreviations, which have also been defined previously. It seems the reviewer may be confusing the figure captions appearing directly after the paragraph in which they are first cited (see comments below), where each abbreviation is defined in the respective figure caption.

**Write as Table(1):---------/Fig.(1):----etc--apply for all**

Response: We followed the guidelines mentioned in the PLOS ONE style templates (PLOSOne_formatting_sample_main_body.pdf), which specify citing figures as “Fig 1”, “Fig 2”, etc., and tables as “Table 1”, “Table 2”, etc.

**There are no figure descriptions**

Response: In accordance with PLOS ONE guidelines, the captions for the 12 figures appear directly after the paragraph in which they are first cited. It seems the reviewer may have mistaken them as part of the main text. Each of the paragraphs starting with Fig X. correspond to a figure caption:

Fig 1: LL 261-265

Fig 2: LL 275-280

Fig 3: LL 299-306

Fig 4: LL 308-316

Fig 5: LL 339-352

Fig 6: LL 378-396

Fig 7: LL 398-407

Fig 8: LL 459-468

Fig 9: LL 470-483

Fig 10: LL 506-512

Fig 11: LL 538-543

Fig 12: LL 561-565

**Results ---**

**Is very long**

Response: The level of detail in the main text is standard for studies describing the histology of the digestive system in fish species. However, since the reviewer may not have recognized the presence of figure captions, they might be assessing the results section as a single text block, including both the main text and figure captions. These captions are positioned throughout the results section after each relevant paragraph (see lines above), in accordance with the journal’s guidelines. Nearly 40% of the section consists of captions for the 12 figures, so the main text of the results section is considerably shorter than what the reviewer may have perceived.

**What about the clinical signs of the treated fishes**

Response: This is a classical nutritional study with a single diet and, as such, it did not involve fish treatments. Clinical signs were not a relevant variable.

**What about the PM changes**

Response: If “PM” refers to postmortem changes, these were not part of our study’s scope nor relevant to its conclusions.

**What about the percentage of mortalities–if there is**

Response: The survival rate is provided in L 259, from which the mortality rate can be easily inferred.

**Resulst and discussion –going to be chapter like**

Response: The discussion section has been rewritten to improve clarity and conciseness.

**Discussion :**

**Rewrite it again**

Response: The discussion section has been revised to reduce its length and improve the flow and clarity.

**Conclusion :**

**Less than enough**

Response: The conclusion sections has been expanded to provide a clearer summary of the findings.

**References:**

**Some cited references need to be more update**

Response: Older references are cited to credit foundational studies. We have reviewed all citations to confirm accuracy and relevance.

**Huge number of references were used (102)???**

Response: The number of references reflects the detail of our study, aligning with PLOS ONE’s policy to include any relevant works. However, the number of references has been reduced to 77 after rewriting the discussion section.

**There are no plan for the study area ?**

Response: Since this is a controlled experiment in an indoor recirculating aquaculture system located in a research institute, there is no geographic study area to map. Instead, the location is provided in the Materials and Methods section in LL 102-103.

**Reviewer #2: Comments to the Authors**

**I have reviewed your manuscript titled "Histological and molecular characterization of the digestive system of early weaned juveniles of Arapaima sp. reared in a recirculating aquaculture system" and would like to offer constructive feedback. I appreciate your effort and dedication and look forward to helping you enhance the quality of your work. Below, I will outline several points that require further consideration and improvement.**

**1. Abstract, Lines 23-29: Please delete or summarize the background information and proceed directly to the aim of the study.**

Response: We have revised the abstract following the reviewer’s comments.

**2. Abstract, Lines 30-35: Please merge the aim of the study to avoid repetition.**

Response: Done

**3. Abstract: Please include specific recommendations in the conclusion to enhance clarity.**

Response: Done. Thank you for this suggestion.

**4. Abstract: In conclusion, please discuss your results and suggest directions for future studies.**

Response: Done. Thank you for this suggestion.

**5. Could you please provide the keywords for this study?**

Response: Keywords were provided during the original submission and not included in the manuscript, as per journal requirements. Reviewer #1 commented on the keywords, so we understand that they are visible to the reviewers. The keywords are: Paiche; Amazon basin; Peruvian aquaculture; digestive gene expression; ontogeny

**6. Introduction, Lines 96-112: The introduction is overly lengthy, and the aim is expressed in too many words. Please revise to clearly emphasize the aim of the study.**

Response: Following your suggestions, we have shortened the introduction and expressed the aim of the study in a more concise manner.

**7. Lines 129 and Table 1: The value of crude lipid differs between the text and Table 1. Please verify and correct this discrepancy.**

Response: Thank you for noticing this inconsistency. The correct value is 15%.

**8. Did you use any anesthetics in the experiment?**

Response: Fish were euthanized with an overdose of eugenol (0.05 μl ml-1; Moyco®,

Moyco, Lima, Peru) (L132-133).

**9. Table 1: All full names of abbreviations, such as gene names, MUST be defined in the footnotes.**

Response: We assume that the reviewer is referring to Table 2. We have now added the full names of all abbreviations in the footnotes as requested

**10. Line 261: Please italicize “(P < 0.05)”. Additionally, check the entire manuscript for consistent formatting.**

Response: Done.

**11. I have noticed a few minor errors and spelling mistakes scattered throughout the manuscript. To enhance the linguistic quality of the manuscript, I would recommend considering professional language revision.**

Response: We have thoroughly revised the manuscript, including a detailed review by one of the co-authors who is a native English speaker.
